# Improving the use of research evidence in guideline development: 13. Applicability, transferability and adaptation

**DOI:** 10.1186/1478-4505-4-25

**Published:** 2006-12-08

**Authors:** Holger J Schünemann, Atle Fretheim, Andrew D Oxman

**Affiliations:** 1 INFORMA/CLARITY Research Group, S.C. Epidemiologia, Istitituto Regina Elena, Via Elio Chianesi 53, 00144 Rome, Italy; 2 Norwegian Knowledge Centre for the Health Services, P.O. Box 7004, St. Olavs plass, N-0130 Oslo, Norway

## Abstract

**Background:**

The World Health Organization (WHO), like many other organisations around the world, has recognised the need to use more rigorous processes to ensure that health care recommendations are informed by the best available research evidence. This is the thirteenth of a series of 16 reviews that have been prepared as background for advice from the WHO Advisory Committee on Health Research to WHO on how to achieve this.

**Objectives:**

We reviewed the literature on applicability, transferability, and adaptation of guidelines.

**Methods:**

We searched five databases for existing systematic reviews and relevant primary methodological research. We reviewed the titles of all citations and retrieved abstracts and full text articles if the citations appeared relevant to the topic. We checked the reference lists of articles relevant to the questions and used snowballing as a technique to obtain additional information. We used the definition "coming from, concerning or belonging to at least two or all nations" for the term international. Our conclusions are based on the available evidence, consideration of what WHO and other organisations are doing and logical arguments.

**Key questions and answers:**

We did not identify systematic reviews addressing the key questions. We found individual studies and projects published in the peer reviewed literature and on the Internet.

Should WHO develop international recommendations?

• Resources for developing high quality recommendations are limited. Internationally developed recommendations can facilitate access to and pooling of resources, reduce unnecessary duplication, and involve international scientists.

• Priority should be given to international health problems and problems that are important in low and middle-income countries, where these advantages are likely to be greatest.

• Factors that influence the transferability of recommendations across different settings should be considered systematically and flagged, including modifying factors, important variation in needs, values, costs and the availability of resources.

What should be done centrally and locally?

• The preparation of systematic reviews and evidence profiles should be coordinated centrally, in collaboration with organizations that produce systematic reviews. Centrally developed evidence profiles should be adaptable to specific local circumstances.

• Consideration should be given to models that involve central coordination with work being undertaken by centres located throughout the world.

• While needs, availability of resources, costs, the presence of modifying factors and values need to be assessed locally, support for undertaking these assessments may be needed to make guidelines applicable.

• WHO should provide local support for adapting and implementing recommendations by developing tools, building capacity, learning from international experience, and through international networks that support evidence-informed health policies, such as the Evidence-informed Policy Network (EVIPNet).

How should recommendations be adapted?

• WHO should provide detailed guidance for adaptation of international recommendations.

• Local adaptation processes should be systematic and transparent, they should involve stakeholders, and they should report the key factors that influence decisions, including those flagged in international guidelines, and the reasons for any modifications that are made.

## Background

The World Health Organization (WHO), like many other organisations around the world, has recognised the need to use more rigorous processes to ensure that health care recommendations are informed by the best available research evidence. This is the thirteenth of a series of 16 reviews that have been prepared as background for advice from the WHO Advisory Committee on Health Research to WHO on how to achieve this.

Adaptation involves modification according to different circumstances or environmental conditions [1]. In the context of guidelines, it relies on judgments of whether a guideline is applicable (i.e. relevant to a local setting in a specific setting) or transferable from one setting to another. A survey of managed care plans in the US found that they relied on national and other published guidelines as references for their own guidelines. However, most of the surveyed plans did not adopt published guidelines "as is" and adapted them for a variety of reasons [2]. The main reasons were lack of local clinical input, inappropriate consideration of resources, failure to apply to a specific population, too extensive recommendations, a high level of complexity in guidelines for users, and failure to include the most recent information in guidelines.

Article II of the World Health Organization (WHO) Constitution defines "setting, validating, monitoring and pursuing the proper implementation of norms and standards" as core functions of the WHO [3]. Accordingly, WHO issues guidelines (for example [4]) that are being used in many countries with the aim of improving the quality of patient care and public health throughout the world [5]. In addition to WHO, an increasing number of organizations develop guidelines. Some of these organizations target international users. For this article we define the term "international" as "concerning or belonging to at least two or all nations". Among organizations that develop guidelines, WHO has the broadest mandate and spectrum of international consumers and stakeholders, given 192 countries are members of WHO and the scope of WHO's responsibilities.

Developing guidelines internationally poses challenges to ensure and monitor that WHO's guidelines are locally applicable or adaptable across different settings. The needs for adapting guidelines identified in the US survey referred to above are even greater for international guidelines. Organisations such as WHO that develop international guidelines need to consider variations in the contexts in which the guidelines will be applied, including differences in needs, values and the availability of resources. In this paper we addressed the following questions:

• Should WHO develop international recommendations?

• What should be done centrally and locally?

• How should recommendations be adapted?

Questions related to guideline implementation and dissemination are specifically addressed in another paper in this series [6].

## What WHO is doing now

The Guidelines for WHO Guidelines (GWG) note that "Governments have as their main responsibility the health of the population, rather than the disease of the individual, and must consider other factors in addition to the traditional concern for maximizing the benefit to individual patients. WHO needs to assess the implications for population health of any recommendation as well. This requires explicit recognition that resources to provide health interventions (including diagnostic procedures, pharmaceuticals, surgical interventions and psychosocial techniques) are limited. This involves considering the cost-effectiveness of alternative interventions, the opportunity costs of investing in one intervention versus another, the affordability of the interventions, and the feasibility of applying a set of recommendations in different settings [7]."

The GWG also recognizes that "WHO takes a global perspective in addressing the needs of (192) member states. Differences in outcome will not only be due to transferring results from a research to a field setting, but also from the different cultural, economic, socio-demographic contexts present in the member-states," and that for "WHO guidelines, the traditional approach of reviewing and reporting evidence on efficacy and safety is certainly crucial but not sufficient. It can be regarded as the first step, but it is also necessary to examine the implications of applying each possible set of recommendations on a population basis. The initial body of evidence to be considered in WHO guidelines will be identical to that of traditional guidelines, but WHO guidelines will need to go further, to take the second step of spelling out the implications of adopting recommendations on costs and on population health. If done adequately, this will allow decision makers in different settings to take the third step of "localizing" the guidelines to their settings, and deciding where the trade-off between additional benefit and additional costs should be set. It will also be useful in determining what is acceptable for the end-users." However, one of the limitations of the GWG is that they have not been operationalized or implemented consistently. Only few WHO guideline processes have followed the GWG [8].

Indeed, the need for international development with local adaptation is expressed in WHO statements such as "The strategy recommends a prevention-oriented approach that emphasizes the need for countries to develop coherent, multi-sectoral national strategies with a long-term, sustainable perspective, to make the healthy choices the preferred alternatives at both the individual and community level. We welcome the commitment shown by Member States to the strategy and will be working closely with them to help them implement its recommendations [9]."

For example, the Global Strategy on Diet, Physical Activity and Health states that "the purpose of the Regional Consultations with Member States is for countries in each region to provide information on the extent of the problem associated with diet, physical activity and chronic disease, and appropriate prevention strategies for their particular countries. The consultation will focus on the discussion of national, regional and international interventions that will be effective within individual countries and that will take account of national, social, cultural and economic realities. Regional differences, common concerns, or international consensus, will be noted and serve as the basis of the development of the Global Strategy. This consultation process will build on past and current activities and programmes on the issue carried out by WHO Regional Offices and by Member States [10]."

WHO also provides funding and support for specific guideline adaptation efforts. Specific WHO guideline adaptation projects exist in the area of HIV that have been supported by international workshops organized by regional offices with country involvement [11,12]. Another example is a WHO-sponsored conference by the International Council of Ophthalmology on local adaptation of clinical practice guidelines in China [13]. However, while WHO is developing international guidelines through a variety of efforts, few WHO groups are using systematic and transparent processes that facilitate judgements regarding their applicability and transferability or provide guidance about how to adapt the guidelines [14].

## What other organisations are doing?

We are not aware of published surveys that address what other organisations do to ensure appropriate adaptation of guidelines. The items that are part of the AGREE instrument [15] include the following three items most relevant for the assessment of guideline applicability:

• The potential organisational barriers in applying the recommendations should be discussed.

• The potential cost implications of applying the recommendations should be considered.

• The guideline should present key review criteria for monitoring and audit purposes.

The Conference on Guideline Standardization (COGS) checklist for reporting clinical practice guidelines suggests that guidelines should: "Describe the intended users of the guideline (e.g., provider types, patients) and the settings in which the guideline is intended to be used" [16]. The checklist does not include any specific recommendations related to supporting judgements about the applicability or transferability of guidelines, or their local adaptation.

There is, however, a growing interest in considerations of how to adapt guidelines [17-22]. This interest is driven by several factors, including a desire to reduce unnecessary duplication of efforts across organisations, limited resources for many organisations, particularly in low and middle-income countries (LMIC), and concerns about the sustainability of programs that are well resourced.

In this background section we provide selected examples of organizations that have specifically provided information relevant to the key questions we posed although the WHO is unique in that its mandate includes the more complex task of providing international guidance.

### SIGN

The Scottish Intercollegiate Guideline Network (SIGN) asks guideline panels to consider issues of applicability when guideline groups summarize their view of the total body of evidence [23]. The guidelines are graded to differentiate between those based on strong evidence and those based on weak evidence. This judgement is made on the basis of a transparent assessment of the design and quality of each study but also a judgement on the consistency, clinical relevance and external validity of the whole body of evidence. The aim is to produce a recommendation that is evidence-based, but which is relevant to the way in which health care is delivered in Scotland and is therefore implementable. The following specific points are included in the described considered judgments SIGN panels are asked to make:

• Generalisability of study findings

• Directness of application to the target population for the guideline.

• Clinical impact (i.e. the extent of the impact on the target patient population, and the resources needed to treat them.)

• Implementability (i.e. how practical it would be for the NHS in Scotland to implement the recommendation.)

SIGN guideline development groups are provided with a form in which to record the main points from their considered judgement [24]. Once they have considered these issues, the group is asked to summarise its view of the evidence and assign a level of evidence to it, before going on to derive a graded recommendation. During this process SIGN guideline developers are also able to downgrade a recommendation if they think the evidence is not generalisable, not directly applicable to the target population, or for other reasons is perceived as being weaker than a simple evaluation of the methodology would suggest. In other areas, the appropriate action may be inclusion in the guideline of a commentary on the main economic issues that should be considered in relation to the subject of the guideline (for example [25]). Another option is the provision of basic information that will allow guideline users to work out the resource implications for their own service (for example [26]).

### New Zealand Guideline Group

**Figure 1:**
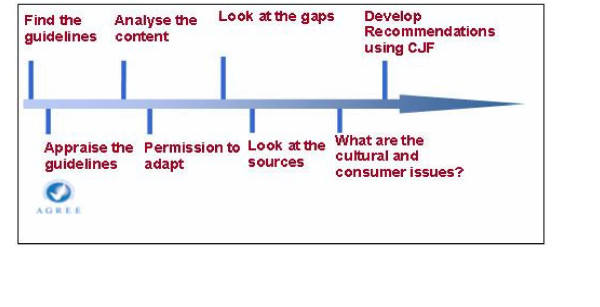
Adapting guidelines using the AGREE instrument (from [23]).

The process recommended by the New Zealand Guideline Group includes the following steps (figure 1) when adapting overseas evidence-based guidelines [27]:

• Appraise the guidelines (using AGREE instrument) for quality and process

• Analyse the content for scope and applicability

∘ Same health settings, professional groups?

∘ Same patients, consumers?

∘ Same interventions?

∘ Same outcomes?

• Look at the gaps in the issues covered in the overseas guidelines

∘ Any clinical questions not covered?

∘ Look at the sources of evidence

∘ Is the search strategy available?

∘ Are there any evidence tables?

• Are the evidence statements and recommendations referenced?

• Re-run the search strategy to include the questions selected

∘ To include literature at least one year prior to the date of publishing

∘ Check if any large study would radically change the recommendations

• Implementation planning

∘ Redesign the implementation plan to meet local circumstances

### Guide to Community Preventive Services

The Guide to Community Preventive Services states the following in a discussion of its methods [28]: "The *Guide *should not be viewed as the sole source for informed public health decision making because local contextual information is also important. Many issues not addressed in the *Guide *will affect which interventions are implemented (e.g., resource availability, social justice, community participation, cultural appropriateness, local burden of diseases and risk factors, and political considerations). However, the *Guide *provides systematically collected and detailed information on several issues of importance to public health practitioners and decision makers; information which is difficult or inefficient to develop locally. *Guide *reviews and recommendations will be most useful in conjunction with a participatory community planning process that clarifies needs and goals and that considers the *Guide*'s evidence reviews and recommendations in conjunction with additional applicable community specific information."

## Methods

The methods used to prepare this review are described in the introduction to this series [29]. Briefly, the key questions addressed in this paper were vetted amongst the authors and the ACHR Subcommittee on the Use of Research Evidence (SURE). For this review we analyzed existing guidelines for guidelines of national or international organizations to identify processes that these organizations use to adapt guidelines locally beyond what was known for existing organizations as described in the background section. We also searched PubMed using "guideline" AND "adaptation OR applicability OR template OR transferability" (MESH headings/keywords) for studies and systematic reviews comparing different strategies to increase adaptation, acceptance and transferability (we identified 637 citations of which 203 citations were identified as systematic reviews using the clinical queries filter for systematic reviews). We reviewed the titles of all citations and retrieved abstracts and full text articles if the citations appeared relevant to the topic. We checked the reference lists of articles relevant to the questions and used snowballing as a technique to obtain additional information. We also searched the Cochrane Library and Google for articles and methods related to guideline adaptation ("guideline adaptation"). In addition, we searched databases maintained by the Agency for Healthcare Research and Quality (AHRQ, [30]) and the Guidelines International Network (GIN, [31]). The answers to the questions are our conclusions based on the available evidence, consideration of what WHO and other organisations are doing and logical arguments.

## Findings

We did not identify systematic reviews addressing the key questions. We found individual studies and projects published in the peer reviewed literature and on the Internet that we will use to illustrate the responses to the key questions.

### Should WHO develop international recommendations?

Threats of new emerging diseases (e.g. severe acute respiratory syndrome (SARS) and avian influenza A (H5N1) infection) as well as pandemics of chronic diseases such as obesity and heart disease have prompted international action and are clear examples of the existence of international health problems and the need for international recommendations. Given the international orientation of WHO and the advantages of large international organizations (e.g. accessing and pooling of resources, reducing unnecessary duplication, and involving international scientists), there is an important role for international recommendations [32]. International recommendations may be most helpful when variation in settings and local circumstances is less important. Therefore, consideration of need (prevalence, baseline risk or health status), setting (e.g. availability of resources) and modifying factors (factors that modify translation of recommendation into practice such as microbiological resistance patterns) can be key components that influence the strength of a recommendation and should be specified in recommendations formulated by the WHO [33].

An illustration of the need for adaptation is provided by Rhinehart and colleagues who attempted to implement a nosocomial infection control program based on the US Centers for Disease Control (CDC) guidelines in an urban Indonesian public hospital [34]. Adoption of unmodified CDC guidelines was impeded by modifying factors such as conditions of the physical plant, absence of an infection control infrastructure, limited sterilization capabilities, lack of clinical microbiologic laboratory support, and the expense of single use medical devices. After on-site evaluations, CDC guidelines were extensively modified so that they were appropriate for local conditions and culture [34]. After implementation, many physical changes had been accomplished, and handling of reusable and disposable medical devises had improved considerably although adoption of clinical practice policies was incomplete.

Global recommendations should apply to most settings yet allow for adaptation to local circumstances. The factors that influence recommendations should be laid out explicitly. If differences in context are likely to lead to different recommendations or decisions, these should be flagged [33,35]. Contextual issues that should be considered include modifying factors, need, values and resources. Table 1 provides a checklist of factors that influence the applicability or transferability of guidelines. It can be used during the guideline development process to help ensure that these factors are considered systematically and transparently, and to clearly label factors that are important to consider in specific settings where the guideline will be applied or adapted.

**Table 1:** Checklist for identifying guidelines requiring adaptation

**Factors influencing the applicability or transferability of guidelines across different settings**	**Response (positive answers increase the likelihood that recommendations should be flagged as requiring adaptation)**
1. Is there important variation in need (prevalence, baseline risk or health status) that might lead to different decisions?	□ Yes □ Unclear □ No
2. Is there important variation in the availability of resources that might lead to different decisions?	□ Yes □ Unclear □ No
3. Is there important variation in costs (e.g. of drugs or human resources) that might lead to different decisions?	□ Yes □ Unclear □ No
4. Is there important variation in the presence of factors that could modify the expected effects (e.g. resistance patterns of microbiological pathogens), which might lead to different decisions?	□ Yes □ Unclear □ No
5. Is there important variation in the relative values of the main benefits and downsides that might lead to different decisions?	□ Yes □ Unclear □ No

### What should be done centrally and locally?

The research evidence on what reflects the best distribution of responsibilities during the development of international guidelines is sparse. Global evidence (i.e. the best evidence from around the world) is the best starting point for judgements about effects and likely modifying factors. Synthesizing and making available this evidence should be coordinated centrally, although the actual work can be done anywhere. For example, the Agency for Health Care and Quality (AHRQ) funds Evidence-based Practice Centres throughout the US and Canada with the methodological competency to undertake systematic reviews, the UK National Institute for Health and Clinical Excellence (NICE) funds National Collaborating Centres responsible for guideline development (in areas such as acute care, cancer and chronic conditions), and the Cochrane Collaboration has 50 Collaborative Review Groups spread around the world that are responsible for preparing and updating systematic reviews (in particular areas such as breast cancer, infectious diseases, and tobacco addiction). WHO could adapt, commission or prepare systematic reviews that are required for guideline development in collaboration with organizations such as these that conduct systematic reviews and follow suggestions to make these reviews more useful for policymakers [36]. Lavis and colleagues suggest that donors and international agencies can encourage more informed public policymaking by supporting national and regional efforts to undertake systematic reviews and assess their local applicability, and by supporting regional or worldwide efforts to coordinate review and assessment processes [37].

Similarly, adaptable evidence profiles [33] based on new or existing high quality systematic reviews that include information on critical outcomes should also be prepared or coordinated centrally. Applying the criteria listed in table 1 and flagging important factors that influence the applicability of guidelines in evidence profiles could facilitate local adaptation and help groups to replace the flagged elements with locally appropriate information. Because resources for guidelines development are limited, particularly in LMIC, support for local adaptation of guidelines should also be supported centrally, by WHO headquarters or by regional offices. Given that WHO also has limited capacity, consideration should be given to doing this collaboratively with other organizations and to developing capacity; e.g. through the development of frameworks and tools, such as those being developed by the International Clinical Epidemiology Network (INCLEN) Knowledge Plus Program [20], GIN [31] and others; through training; through networks such as the Evidence-Informed Policy Network (EVIPNet) [38], and by learning from the experience of organizations around the world that are engaged in supporting evidence-informed health policies in specific settings [39].

Apparently successful examples of collaboration between central and country level groups exist. Wabitsch and colleagues described that during the adaptation of the WHO global HIV/AIDS guidelines [11] standard techniques involving consensus building were successfully employed to adapt these guidelines to local settings (Malawi and Barbados). The results showed that the process preserved the structure but involved significant modification to the processes of clinical care. Given the factors that influence the formulation of recommendations, the modification of individual recommendations confirms that an adaptation process was required.

### How should recommendations be adapted?

In addition to supporting appropriate adaptation of its own guidelines, WHO should consider adapting guidelines developed by other organizations, given the potential value of WHO endorsement and savings, if high quality guidelines already exist. Detailed guidance on appropriate methods for adapting guidelines would help WHO guideline groups to adapt existing guidelines, when this is appropriate. Adaptation of recommendations is required because several judgments influence recommendations. Therefore, recommendations dealing with identical questions may differ between developers despite reliance on the same evidence. Implementation, which follows the process of adaptation, is topic of another paper in this series and tools for the evaluation of implementation of guidelines have been developed [6,40].

**Figure 2:**
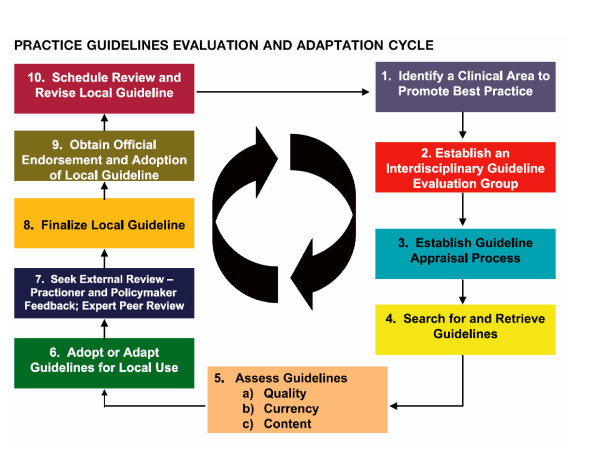
The Practice Guideline Evaluation and Adaptation Cycle (PGEAC) (from [17, 41]).

Decisions during local adaptation processes should be transparent and follow procedures that are similar to those used in developing the guidelines, including reporting the key factors that influence any modifications. Two fairly similar approaches have recently appeared that produced frameworks for identifying candidate guidelines for local adaptation. The Practice Guideline Evaluation and Adaptation Cycle (PGEAC) is a 10 step approach (figure 2) [17,41,42]. Graham and colleagues describe three alternatives in the PGEAC approach: (a) adopt one guideline with all its recommendations; (b) adopt one guideline, endorsing some of its recommendations but not endorsing recommendations that lack strong evidence or cannot be implemented or adapted locally; or (c) take the best recommendations from each of the guidelines and adapt them for inclusion of the new guideline [17]. If recommendations are modified the rationale for changes should be explicitly stated in the resulting local guideline document.

The other approach has been developed by the international working group ADAPTE [18,19,22] and partly overlaps with the PGEAC approach. Recently the groups developing both adaptation approaches merged with the purpose of developing a generic manual on guideline adaptation. The manual will undergo pilot testing. The group is calling itself the ADAPTE group . Whatever adaptation process is chosen the process should be made explicit, undergo review by peers, and involve consumers, policymakers and other stakeholders who may provide input about policy guidance.

Balance sheets or evidence profiles are designed to assist decision-makers regarding outcomes in their practice setting [43,44]. For guideline adaptation they should include data for the specific setting to which they are adapted (for all the considerations in table 1). During adaptation consumer involvement (i.e. to integrate their values and perspectives) and involvement of other stakeholders should be emphasized [35,45].

## Discussion

Given WHO's mandate; limited resources that are available to develop high quality guidelines that are informed by the best available evidence, particularly in LMIC; and the potential to reduce unnecessary duplication, WHO should continue to develop international guidelines. However, these guidelines will often require adaptation and tailoring to local contexts and WHO should, so far as possible, provide support to help ensure that international guidelines are adapted appropriately to local circumstances. To do this WHO must ensure that it systematically considers needs for local adaptation when developing guidelines; and that it has sufficient capacity to support both developing and supporting the adaptation of high priority guidelines. To do this as effectively and efficiently as possible WHO should collaborate with other organizations both in developing guidelines and, importantly, in developing capacity in LMIC and supporting appropriate adaptation in countries that lack resources. We provide other recommendations about how WHO can improve the implementation of organizational changes to guideline development in other articles in this series [6,46-50].

### Further work

WHO has ample experience in adapting guidelines but this effort should be coordinated and disseminated among WHO guideline groups. Through coordinating guideline development within WHO and collaborating with other organizations, WHO could capitalise on this experience, improve the quality of its guidelines, and help to ensure that its guidelines are appropriately adapted and result in appropriate actions and health improvements. Similar suggestions have been made previously by investigators involved with WHO guideline projects. For example, "Countries should discuss and find ways of collaboration and formation of linkages and support with National HIV/AIDS program in order to enhance the implementation of IMCI algorithm which includes HIV/AIDS" [11].

A systematic review of studies evaluating methods for adaptating guidelines is unlikely to retrieve high quality evidence given the paucity of research in this area. Further development and evaluation of frameworks and tools to support the appropriate adaptation of guidelines is needed. Given the limited capacity for this in many countries, comparisons of simpler processes that require fewer resources should be compared with more rigorous processes to determine the most efficient methods for ensuring that guidelines support well-informed decisions and actions appropriate for the specific contexts in which they are taken.

## Competing interests

ADO and AF work for the Norwegian Knowledge Centre forthe Health Services, an agency funded by the Norwegian government that produces systematic reviews and health technology assessments. All three authors are contributors to the Cochrane Collaboration. ADO and HJS are members of the GRADE Working Group. HJS is documents editor and chair of the documents development and implementation committee for the American Thoracic Society and senior editor of the American College of Chest Physicians' Antithrombotic and Thrombolytic Therapy Guidelines.

## Authors' contributions

HJS prepared the first draft of this review. AF and ADO contributed to drafting and revising it. All authors read and approved the final manuscript.
